# Outcomes of neonates born following transfers of frozen-thawed cleavage-stage embryos with blastomere loss: a prospective, multicenter, cohort study

**DOI:** 10.1186/s12916-018-1077-8

**Published:** 2018-06-19

**Authors:** Yan-Ting Wu, Cheng Li, Yi-Min Zhu, Shu-Hua Zou, Qiong-Fang Wu, Li-Ping Wang, Yan Wu, Rong Yin, Chao-Yi Shi, Jing Lin, Zi-Ru Jiang, Yi-Jing Xu, Yun-Fei Su, Jian Zhang, Jian-Zhong Sheng, William D. Fraser, Zhi-Wei Liu, He-Feng Huang

**Affiliations:** 1 International Peace Maternity and Child Health Hospital, School of Medicine, Shanghai Jiao Tong University, No. 910 Hengshan Rd, Shanghai, 200030 China; 2 Institute of Embryo-Fetal Original Adult Diseases, Shanghai Jiao Tong University School of Medicine, Shanghai, China; 3 Department of Reproductive Endocrinology, Women’s Hospital, School of Medicine, Zhejiang University, Zhejiang, China; 4 Department of Reproductive Medicine, Qingdao Women and Children’s Hospital, Shandong, China; 5 Department of Reproductive Medicine, Jiangxi Maternal and Child Health Hospital, Jiangxi, China; 6 Department of Reproductive Medicine, Jiaxing Maternity and Child Health Care Hospital, Zhejiang, China; 7 Department of Pathology and Pathophysiology, School of Medicine, Zhejiang University, Zhejiang, China; 8 Key Laboratory of Reproductive Genetics, Ministry of Education (Zhejiang University), Zhejiang, China; 9 Centre de recherche du Centre Hospitalier Universitaire de Sherbrooke (CRCHUS) and Department of Obstetrics and Gynecology, University of Sherbrooke, Sherbrooke, QC, Canada

**Keywords:** Frozen embryo transfer, Blastomere loss, Pregnancy outcomes, Small for gestational age, Transient tachypnea of the newborn, Cohort study

## Abstract

**Background:**

Despite limited information on neonatal safety, the transfer of frozen-thawed cleavage-stage embryos with blastomere loss is common in women undergoing in vitro fertilization. We aimed to evaluate the pregnancy outcomes and safety of frozen-thawed cleavage-stage embryos with blastomere loss.

**Methods:**

This prospective, multicenter, cohort study included all frozen-thawed cleavage-stage embryo transfer (FET) cycles between 2002 and 2012. Pregnancy outcomes and subsequent neonatal outcomes were compared between FET cycles with intact embryos and those with blastomere loss.

**Results:**

A total of 12,105 FET cycles were included in the analysis (2259 cycles in the blastomere loss group and 9846 cycles in the intact embryo group). The blastomere loss group showed significantly poorer outcomes with respect to implantation, pregnancy, and live birth rates than the intact embryo group. However, following embryo implantation, the two groups were similar with respect to live birth rates per clinical pregnancy. Among multiple pregnancies (4229 neonates), neonates from the blastomere loss group were at an increased risk of being small for gestational age (aOR = 1.50, 95% CI 1.00–2.25) compared to those from the intact group. A similar trend was observed among singletons (aOR = 1.84, 95% CI 0.99–3.37). No associations were found between blastomere loss and the subsequent occurrence of congenital anomalies or neonatal mortality. However, neonates from the blastomere loss group were at an increased risk of transient tachypnea of the newborn (aOR = 5.21, 95% CI 2.42–11.22).

**Conclusions:**

The transfer of embryos with blastomere loss is associated with reduced conception rates. Once the damaged embryos have implanted, pregnancies appear to have the same probability of progressing to live birth but with an increased risk of small for gestational age neonates and transient tachypnea of the newborn.

**Study registration:**

This study was retrospectively registered at Chinese Clinical Trial Registry. Registration number: ChiCTR-OOC-16007753. Registration date: 13 January 2016.

**Electronic supplementary material:**

The online version of this article (10.1186/s12916-018-1077-8) contains supplementary material, which is available to authorized users.

## Background

Embryo cryopreservation with subsequent frozen-thawed embryo transfer (FET) has been increasingly used in assisted reproductive technology since it was first reported in 1984 [1]. The benefits of FET include improved cumulative pregnancy rates, a reduced risk of adverse outcomes related to multiple gestations, and a lower incidence of ovarian hyperstimulation syndrome [2, 3]. As FET has become an essential part of assisted reproductive technology, the number of FET cycles has increased over the last decade [4, 5]. However, concerns have been raised regarding the safety of FET, particularly in terms of pregnancy and perinatal outcomes.

Blastomere loss among cryopreserved embryos is a common phenomenon following the cryopreservation and thawing process with the alterations in osmotic pressure. Findings are conflicting regarding perinatal outcomes following transfer cycles of cleavage-stage embryos with blastomere loss. Several studies have revealed that the transfer of embryos with blastomere loss results in implantation rates that are comparable to those following the transfer of embryos with intact blastomeres [6–8]. However, other studies have reported that blastomere loss might be associated with a reduction in the implantation rate, impaired in vitro development after thawing and impaired embryo survival following embryo transfer [9–13]. Although embryos with blastomere loss can be transferred and can implant and subsequently develop, the safety for the offspring is a concern for researchers and clinicians.

To address this knowledge gap, we conducted a multicenter cohort study aiming to assess the impact of the transfer of embryos with blastomere loss relative to that of intact embryos on pregnancy outcomes following FET, including safety outcomes for newborn infants.

## Methods

### Study design and participants

This prospective cohort study included all infertile women undergoing FET between 2002 and 2012 in five centers in China. To investigate the impact of blastomere loss on pregnancy outcomes and safety for neonates, only cases with embryos that were cryopreserved at the cleavage-stage were included in this study. Mixed transfers in which intact embryos and embryos with blastomere loss were transferred together were excluded. Furthermore, patients who received donated oocytes or sperm or who underwent preimplantation genetic diagnosis (PGD) or preimplantation genetic screening due to an increased risk of inherited diseases were also excluded. Women with blastocyst transfers in FET cycles were beyond the scope of this study.

Institutional Review Board approval for the study was obtained to conduct the research in the participating centers, including International Peace Maternity and Child Health Hospital, Women’s Hospital of Zhejiang University, Qingdao Women and Children’s Hospital, Jiangxi Maternal and Child Health Hospital, and Jiaxing Maternity and Child Health Care Hospital. Written informed consent forms were obtained from all participants before inclusion. The reporting of this study conforms to the STROBE statement.

### Procedures

The process of in vitro fertilization (IVF) was conducted per routine methods including ovarian stimulation, oocyte retrieval, and insemination by either conventional IVF or intracytoplasmic sperm injection. Embryo cryopreservation was performed by slow freezing or vitrification, as previously described [14, 15], due to either (1) a maternal condition that was unsuitable for fresh embryo transfer, such as a high estrogen level, ovarian hyperstimulation syndrome, or a desynchronized endometrium or (2) when embryos had been harvested in a previous, unsuccessful IVF cycle. Before embryo thawing, the endometrium was prepared by natural monitoring, an ovarian stimulation cycle, or hormone replacement therapy, with estrogen based on clinical indications [16]. After excluding cases in which transfers were performed with mixed embryos (including both intact embryos and embryos with blastomere loss), transfers were dichotomized as transfer with embryos having lost at least one blastomere (blastomere loss group) or transfer with intact embryos (intact embryo group). Generally, embryos losing more than 50% of their original blastomere are not suitable for transfer, so embryos transferred in this study had at least 50% of their blastomeres [17].

Sociodemographic characteristics, reproductive history, and the smoking status of the women and their partners were documented using a questionnaire that was administered by clinical personnel.

Pregnancy outcome measures following FET were assessed at follow-up visits at study medical centers. Ultrasound scans and the serum β-hCG level were part of the routine clinical care. The results of all clinically indicated scans were reported, and paper copies were filed in both hospital case records and the participant’s hand-held notes.

Pregnancy outcomes following all FET cycles were assessed and included the implantation rate, chemical pregnancy rate, clinical pregnancy rate, ongoing pregnancy rate, live birth rate per transfer cycle, and live birth per clinical pregnancy. The implantation rate was defined as the number of gestational sacs measured by ultrasound relative to the number of embryos transferred in the transfer cycle. The chemical pregnancy rate was defined as the proportion of women with a serum β-hCG of more than 10 mIU/mL following FET. Clinical pregnancy was defined as a pregnancy documented by ultrasound at 6–8 gestational weeks that showed a gestational sac inside the uterus, and the clinical pregnancy rate was calculated using the number of clinical pregnancies divided by the number of FET cycles. Ongoing pregnancy was defined as a pregnancy documented by ultrasound at 12 gestational weeks that showed the presence of a fetal heartbeat. The ongoing pregnancy rate was calculated using the number of ongoing pregnancies divided by the number of FET cycles. A live birth was defined as the delivery of one or more infants with any signs of life after 28 weeks of gestation, and the live birth rate was calculated on the basis of the number of FET cycles and clinical pregnancies. Furthermore, adverse outcomes including early miscarriage before 12 gestational weeks, stillbirth, ectopic pregnancy, and pregnancy termination due to fetal defects were also recorded.

Maternal pregnancy complications were collected to evaluate the effects of blastomere loss on obstetric and neonatal outcomes. The pregnancy complication variables documented included gestational diabetes, hypertensive disorders during pregnancy, intrahepatic cholestasis of pregnancy, preterm delivery, and mode of delivery.

The neonatal outcome assessments included birthweight for gestational age, Apgar score at 1 and 5 min, neonatal respiratory disorders, congenital anomalies, and neonatal mortality. Details were abstracted from hospital records within 1 month of delivery. Small for gestational age (SGA) was defined as a birthweight below the 10th percentile for the gestational age, and large for gestational age was defined as a birthweight over the 90th percentile [18]. Preterm birth was defined as birth prior to 37 weeks of gestation. Neonatal respiratory disorders, including transient tachypnea of the newborn (TTN) and neonatal respiratory distress syndrome (RDS), were abstracted from hospital records. A congenital anomaly was defined as a deformity and/or developmental abnormality of any organ or system. Neonatal mortality was defined as infant death during the first 28 days of life.

### Statistical analysis

Continuous variables are expressed as the means ± standard deviation, and the differences in the continuous variables between groups were tested with the *t* test or Mann–Whitney U test. Categorical variables are represented as frequencies with proportions, and differences were detected with the Pearson χ^2^ test or Fisher’s exact test, as appropriate.

Neonatal outcomes following FET were stratified according to singleton or multiple deliveries. To study the associations between blastomere loss and adverse neonatal outcomes, odds ratios (ORs) were calculated for each outcome using logistic regression. To analyze the neonatal outcomes of singletons, multivariable logistic regression was used, and ORs and 95% confidence intervals (CIs) were adjusted for the potential confounding factors including the type of embryo cryopreservation, gestational diabetes mellitus, hypertensive disorder, paternal smoking during pregnancy, number of previous abortions, primary infertility, preterm delivery, and mode of delivery. When we analyzed the neonatal outcomes of multiples, odds ratios (ORs) and 95% confidence intervals (CIs) were obtained using multilevel logistic regression and adjusted for the same confounding factors as for singletons, according to Carlin et al. [19].

All statistical analyses were performed using the SAS software, version 9.3 (SAS Institute, Inc., Cary, NC, USA). All *p* values were calculated using two-sided tests, and differences were considered significant if the *p* value was less than 0.05.

## Results

**Fig. 1:**
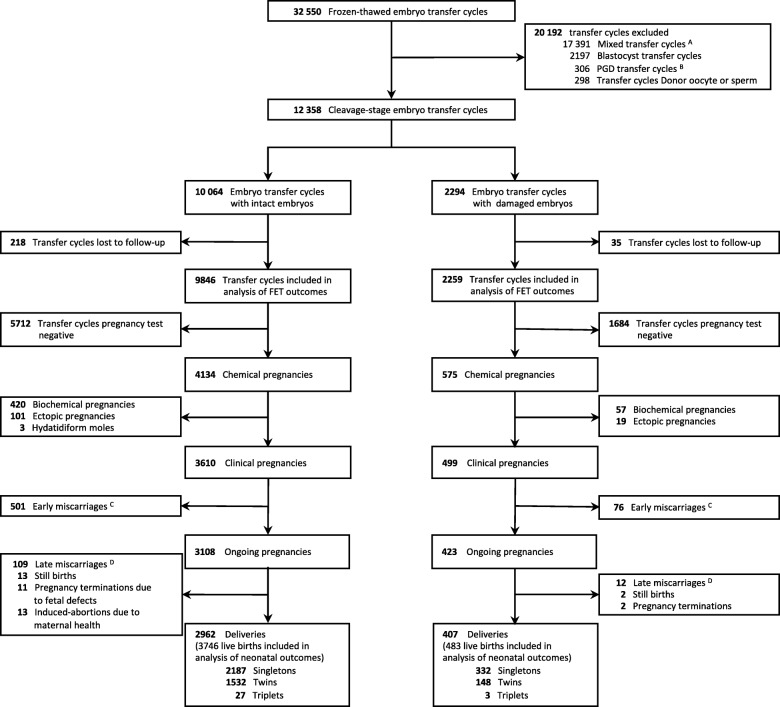
Study flow chart. *A* Mixed transfer cycle was defined as transferring more than one embryo with the transfer comprising an intact embryo and an embryo with blastomere loss. *B* PGD preimplantation genetic diagnosis. *C* Early miscarriage was defined as spontaneous loss of pregnancy before 12 gestational weeks. *D* Late miscarriage was defined as pregnancy loss between 12 and 28 gestational weeks

Figure 1 outlines the flow of the study participants. A total of 32,550 FET cycles were identified at five medical centers from 2002 to 2012. Mixed transfer cycles, blastocyst transfer cycles, patients who received donated oocytes or sperm, and patients who underwent PGD due to inherited diseases were excluded. In total, 12,358 transfer cycles met the inclusion criteria. After excluding 253 transfer cycles that were lost to follow-up or in patients who refused to participate, a total of 12,105 transfer cycles were included in the analysis (9846 transfer cycles in the intact embryo group and 2259 transfer cycles in the blastomere loss group), with a follow-up rate of 98.0%. Women with live born newborns were included in the neonatal outcomes analysis. The ratio of singletons to multiples was 1.40 (2187 to 1559 neonates) in the intact embryo group and 2.20 (332 to 151 neonates) in the blastomere loss group.

The maternal age at oocyte retrieval, maternal body mass index, proportion of multiparity, duration of infertility, and distribution of the causes of infertility were comparable between groups (Table 1). Differences in the age in days at embryo transfer and the type of FET cycle were small and unlikely to be clinically significant (Table 1). Embryo cryopreservation by slow freezing was more frequent in the blastomere loss group than in the intact embryo group.

**Table 1:** Baseline characteristics of all transfer cycles

	Intact embryo group (*n* = 9846)	Blastomere loss group (*n* = 2259)	*p* value
	No. (%)	No. (%)	
History of reproduction			
Age of oocyte retrieval, mean (SD), years	31.12 (4.57)	31.18 (4.05)	0.515
Age of embryo transfer, mean (SD), years	31.64 (4.59)	31.84 (4.13)	0.046
BMI, mean (SD), kg/m^2^	21.76 (3.00)	21.68 (4.35)	0.409
Duration of infertility, mean (SD), years	4.31 (2.91)	4.42 (3.00)	0.114
Pluriparous	731 (7.4)	176 (7.8)	0.550
Primary infertility	5048 (51.3)	1265 (56.0)	< 0.001
Causes of infertility			
Tubal infertility	5265 (53.5)	1234 (54.6)	0.188
Anovulatory	205 (2.1)	56 (2.5)	
Endometriosis	201 (2.0)	57 (2.5)	
Male-factor infertility	1721 (17.5)	365 (16.2)	
Unexplained infertility	330 (3.4)	63 (2.8)	
Combined ^a^	2124 (21.6)	484 (21.4)	
Characteristics of FET cycle			
Type of embryo cryopreservation			
Slowing freezing	3986 (40.5)	1793 (79.4)	< 0.001
Vitrification	5860 (59.5)	466 (20.6)	
Type of FET cycle			
Natural	5085 (51.7)	1255 (55.6)	0.004
HRT	3063 (31.1)	654 (28.6)	
OS	1698 (17.3)	359 (15.9)	
Numbers of embryo transferred			
1	803 (8.2)	320 (14.2)	< 0.001
2	6679 (67.8)	1015 (44.9)	
3	2364 (24.0)	924 (40.9)	

*BMI* body mass index, *FET* frozen-thawed embryo transfer, *HRT* hormone replacement therapy, *OS* ovarian stimulation

^a^Combined was defined as two or more infertile causes mentioned above

Table 2 shows the pregnancy outcomes per transfer cycle for the two study groups. The implantation rate was significantly higher in the intact embryo group than in the blastomere loss group (21.8% vs. 12.7%, *p* < 0.001). Additionally, the intact embryo group showed higher rates of chemical pregnancies (42.0% vs. 25.5%, *p* < 0.001), clinical pregnancies (36.7% vs. 21.9%, *p* < 0.001), ongoing pregnancies (31.6% vs. 18.7%, *p* < 0.001), and live births (30.1% vs. 18.0%, *p* < 0.001) per transfer cycle than the blastomere loss group. However, the live birth rate per clinical pregnancy showed no significant difference between the two groups (82.1% vs. 81.6%, *p* = 0.791). The blastomere loss group was similar to the intact embryo group with respect to the ectopic pregnancy rate (1.0% vs. 0.8%, *p* = 0.424), early pregnancy loss rate (13.9% vs. 15.2%, *p* = 0.415), stillbirth rate (0.4% vs. 0.4%, *p* = 0.703), and pregnancy termination rate due to the fetal defects (0.3% vs. 0.4%, *p* = 0.666). Similar findings were observed in the stratified analysis performed according to the number of embryos transferred and the type of cryopreservation (shown in Additional files 1 and 2). Furthermore, the stratified analysis performed according to the number of embryos transferred showed a lower clinical pregnancy rate and live birth rate per transfer cycle in the blastomere loss group. However, similar live birth rates per clinical pregnancy were observed between the two groups (Additional files 3 and 4).

**Table 2:** Pregnancy outcomes following transferring embryos with or without blastomere loss

	Intact embryo group (*n* = 9846)	Blastomere loss group (*n* = 2259)	*p* value
Total no. of embryo transferred	21,136	5036	
Embryo implantation rate (*n*, %)^a^	4615 (21.8)	641 (12.7)	< 0.001
Chemical pregnancies (*n*, %)^b^	4134 (42.0)	575 (25.5)	< 0.001
Clinical pregnancies (*n*, %)^c^	3610 (36.7)	499 (22.1)	< 0.001
Ongoing pregnancies (*n*, %)^d^	3108 (31.6)	423 (18.7)	< 0.001
Live births (*n*, % per embryo transfer cycle)^e^	2962 (30.1)	407 (18.0)	< 0.001
Live births (n, % per clinical pregnancy)^f^	2962 (82.1)	407 (81.6)	0.791
Singleton (% per live birth)	2187 (73.8)	332 (81.6)	0.003
Twins (% per live birth)	76 (25.9)	74 (18.2)	
Triplets (% per live birth)	9 (0.3)	1 (0.3)	
Ectopic pregnancies (*n*, %)	101 (1.0)	19 (0.8)	0.424
Early miscarriages (*n*, % per clinical pregnancy)	501 (13.9)	76 (15.2)	0.415
Stillbirths (*n*, % per clinical pregnancy)	13 (0.4)	2 (0.4)	0.703
Pregnancy termination due to fetal anomaly (n, % per clinical pregnancy)^g^	11 (0.3)	2 (0.4)	0.666

^a^The implantation was defined as an observation of gestational sacs by ultrasound. The implantation rate was defined as the number of gestational sacs divided by the number of embryos transferred

^b^Chemical pregnancy was defined as an elevated serum β-hCG level of more than 10 mIU/mL. Chemical pregnancy rate was defined as the number of chemical pregnancies divided by the number of embryo transfer cycles for each group

^c^Clinical pregnancy was defined as a pregnancy documented by ultrasound at 6–8 gestational weeks that showed a gestational sac inside the uterus. Clinical pregnancy rate was defined as the number of clinical pregnancies divided by the number of embryo transfer cycles for each group

^d^Ongoing pregnancy was defined as a pregnancy documented by ultrasound at 12 gestational weeks that showed the presence of fetal heartbeat. Ongoing pregnancy rate was defined as the number of ongoing pregnancies divided by the number of embryo transfer cycles for each group

^e^Live birth was defined as the delivery of one or more infants with any signs of life after 28 gestational weeks. Live birth rate (% per embryo transfer cycle) was defined as the number of live birth divided by the number of embryo transfer cycle for each group

^f^Live birth rate (% per clinical pregnancy) was defined as the number of live birth divided by the number of clinical pregnancy for each group

^g^Of the 11 patients who terminated the pregnancy in the intact embryo group, three were diagnosed as chromosome anomalies, two were never system development disorder, two were multiple malformation, the rest were congenital heart disease, umbilical hernia, bilateral renal agenesis, and achondroplasia. Both patients in the blastomere loss group terminated pregnancy due to limb deformity

Table 3 reports the association between the percentage of blastomere loss and the pregnancy outcomes following single FET. The chemical pregnancy rate (*p*_for trend_ = 0.011), clinical pregnancy rate (*p*_for trend_ = 0.011), ongoing pregnancy rate (*p*_for trend_ = 0.032), and live birth rate (*p*_for trend_ = 0.020) decreased progressively in relation to the percentage of blastomere loss. Interestingly, where blastomere loss was less than 25%, no significant differences were evident between intact embryos and embryos with blastomere loss in terms of the chemical pregnancy rate, clinical pregnancy rate, ongoing pregnancy rate, or live birth rate per transfer cycle.

**Table 3:** Pregnancy outcomes related to the percentage of blastomere loss following single frozen-thawed embryo transfer

	Percentage of blastomere loss			*p*_*1*_^a^	*p*_*2*_^a^	*p*_*3*_^a^	*p*_*for trend*_^b^
	0% (*n* = 803)	≤ 25% (*n* = 154)	26–50% (*n* = 166)				
Chemical pregnancies (n, %)^c^	169(21.1)	35 (22.7)	18 (10.8)	0.641	0.002	0.004	0.018
Clinical pregnancies (n, %)^d^	145 (18.1)	28 (18.2)	15 (9.0)	0.971	0.004	0.017	0.020
Ongoing pregnancies (n, %)^e^	120 (14.9)	26 (16.9)	12 (7.2)	0.540	0.008	0.008	0.021
Live births (n, % per embryo transfer cycle)^f^	114 (14.2)	25 (16.2)	10 (6.0)	0.511	0.004	0.004	0.041
Live births (n, % per clinical pregnancy)^g^	114 (77.1)	25 (89.3)	10 (66.7)	0.194	0.331	0.104	0.950
Ectopic pregnancies (n, %)	4 (0.5)	0 (0.0)	0 (0.0)	0.380	1.000	NA	0.219
Early miscarriages (n, % per clinical pregnancy)	25 (17.2)	2 (7.7)	3 (17.7)	0.257	0.728	0.324	0.503
Pregnancy termination due to fetal anomaly (*n*, % per clinical pregnancy) ^h^	1 (0.7)	0 (0.0)	0 (0.0)	1.000	1.000	NA	0.531

*NA* not accessible

^a^*P* values were calculated using Pearson χ^2^ test or Fisher exact test, *p*_*1*_ for comparisons between 0% and ≤ 25% group, *p*_*2*_ for comparisons between 0% and 26–50% group, *p*_*3*_ for comparisons between ≤ 25% and 26–50% group

^b^*P*_for trend_ was calculated using Cochran–Mantel–Haenszel test, and adjusted for primary infertility and the type of FET cycle

^c^Chemical pregnancy was defined as an elevated serum β-hCG level of more than 10 mIU/mL. Chemical pregnancy rate was defined as the number of chemical pregnancies divided by the number of embryo transfer cycle for each group

^d^Clinical pregnancy was defined as a pregnancy documented by ultrasound at 6–8 gestational weeks that showed a gestational sac in the uterus. Clinical pregnancy rate was defined as the number of clinical pregnancies divided by the number of embryo transfer cycles for each group

^e^Ongoing pregnancy was defined as a pregnancy documented by ultrasound at 12 gestational weeks that showed the presence of fetal heartbeat. Ongoing pregnancy rate was defined as the number of ongoing pregnancies divided by the number of embryo transfer cycles for each group

^f^Live birth was defined as the delivery of one or more infants with any signs of life after 28 gestational weeks. Live birth rate (% per embryo transfer cycle) was defined as the number of live births divided by the number of embryo transfer cycles for each group

^g^Live birth rate (% per clinical pregnancy) was defined as the number of live births divided by the number of clinical pregnancies for each group

^h^One patient in 0% group terminated the pregnancy due to achondroplasia

Table 4 presents the maternal demographic characteristics, reproductive history, and pregnancy complications according to study group. There was no statistical evidence of a difference in pregnancy complications between the groups in terms of hypertensive disorders, intrahepatic cholestasis of pregnancy, and preterm delivery. Cesarean section was less likely in the intact embryo group (84.6% vs. 89.8%, *p* = 0.005), but no difference in the delivery method was found between the groups for multiple deliveries (97.9% vs. 96.0%, *p* = 0.231, data not shown in the table).

**Table 4:** Maternal characteristics and pregnancy complications of pregnancies carried to delivery after transferring embryos with or without blastomere loss

	Intact embryo group (*n* = 2962)	Blastomere loss group (*n* = 407)	*p* value
	No. (%)	No. (%)	
Maternal socio-demographic characteristics			
Pre-gestational BMI, mean (SD), kg/m^2^	21.79 ± 2.92	21.63 ± 2.66	0.265
Education attainment			
Primary school or lower	104 (3.5)	19 (4.7)	0.487
Middle or high school	1524 (51.5)	204 (50.1)	
Collage or above	1334 (45.0)	184 (45.2)	
Maternal smoking during pregnancy	5 (0.2)	1 (0.3)	0.538
Paternal smoking during pregnancy	379 (12.8)	71 (17.4)	0.010
History of reproduction			
Parity	164 (5.5)	32 (7.9)	
1	155 (5.2)	30 (7.4)	0.109
≥ 2	9 (0.3)	2 (0.5)	
Number of previous abortion			
0	1597 (53.9)	184 (45.2)	0.004
1–2	1095 (37.0)	180 (44.2)	
≥ 3	270 (9.1)	43 (10.6)	
Previous ectopic pregnancy			
No	2935 (99.1)	403 (99.0)	0.784
Yes	27 (0.9)	4 (1.0)	
Duration of infertility, year			
≤ 2	863 (29.1)	125 (30.7)	0.795
3–4	1008 (34.0)	137 (33.7)	
≥ 5	1091 (36.8)	145 (35.6)	
Primary infertility			
No	1555 (52.5)	179 (44.0)	0.001
Yes	1407 (47.5)	228 (56.0)	
Causes of infertility			
Tubal infertility	1625 (54.9)	222 (54.6)	0.633
Anovulatory	70 (2.4)	12 (3.0)	
Endometriosis	60 (2.0)	8 (2.0)	
Male-factor infertility	549 (18.5)	78 (19.2)	
Unexplained infertility	87 (2.9)	6 (1.5)	
Combined^a^	571 (19.3)	81 (19.9)	
Type of embryo cryopreservation			
Slowing freezing	1037 (35.0)	318 (78.1)	< 0.001
Vitrification	1925 (65.0)	89 (21.9)	
Type of FET cycle			
Natural	1559 (52.6)	216 (53.1)	0.435
HRT	858 (29.0)	126 (31.0)	
OS	545 (18.4)	65 (16.0)	
Pregnancy complications			
Gestational diabetes mellitus			
No	2773 (93.6)	383 (94.1)	0.707
Yes	189 (6.4)	24 (5.9)	
Hypertensive disorder			
No	2525 (85.3)	349 (85.8)	0.994
Gestational hypertension	201 (6.8)	27 (6.6)	
Mild pre-eclampsia	166 (5.6)	22 (5.4)	
Wild pre-eclampsia	70 (2.4)	9 (2.2)	
Intrahepatic cholestasis of pregnancy			
No	2856 (96.4)	388 (95.3)	0.276
Yes	106 (3.6)	19 (4.7)	
Amniotic fluid abnormality			
No	2842 (96.0)	388 (95.3)	0.835
Hydramnios	46 (1.6)	7 (1.7)	
Oligohydramnios	74 (2.5)	12 (3.0)	
Preterm delivery ^b^			
No	2323 (78.4)	326 (80.1)	0.741
Preterm	546 (18.4)	69 (17.0)	
Very preterm	93 (3.1)	12 (3.0)	
Mode of delivery			
Vaginal	456 (15.4)	41 (10.1)	0.005
Cesarean section	2506 (84.6)	366 (89.9)	

*FET* frozen-thawed embryo transfer, *BMI* body mass index, *HRT* hormone replacement therapy, *OS* ovarian stimulation, *NA* not accessible

^a^Combined was defined as two or more infertile causes mentioned above

^b^Preterm was defined as delivery prior to 37 gestational weeks, and very preterm was defined as delivery between 28 and 32 gestational weeks

A multivariable analysis of neonatal outcomes stratified according to multiplicity (singleton and multiple deliveries) is shown in Table 5. The sexes of the neonates were similar between groups. There was no evidence of differences between the study group in terms of low birthweight (singletons: adjusted OR (aOR) = 1.42, 95% CI 0.76–2.66; multiple births: aOR = 1.07, 95% CI 0.71–1.61) or macrosomia (singletons: aOR = 1.08, 95% CI 0.72–1.62; no macrosomia in multiple births). However, among multiple births, neonates born from embryos with blastomere loss showed a trend toward an increased risk of SGA birth (aOR = 1.50, 95% CI 1.00–2.25). For singletons, a similar trend was observed (aOR = 1.84, 95% CI 0.99–3.37). Multiple births did not change the risk of large for gestational age (singletons: aOR = 0.80, 95% CI 0.57–1.13; multiple births: aOR = 0.44, 95% CI 0.10–1.90). Neonates born from embryos with blastomere loss had a significantly increased risk of TTN compared with those born from intact embryos (singletons: aOR = 6.27, 95% CI 1.86–21.14; multiple births: aOR = 5.21, 95% CI 1.90–14.27); however, there was no evidence of a difference between groups in the risk of RDS (singletons: aOR = 1.11, 95% CI 0.38–3.29; multiple births: aOR = 0.77, 95% CI 0.37–1.60). No association was found between neonates born from embryos with blastomere loss or the Apgar score at 1 or 5 min. In total, 23 neonates were born with congenital anomalies (20/3746 in the intact embryo group and 3/483 in the blastomere loss group), and no risk was observed between congenital anomalies and blastomere loss (aOR = 1.18, 95% CI 0.32–4.31). The neonatal mortality rate was similar between groups (0.2% (8/3746) in the intact embryo group and 0.2% (1/483) in the blastomere loss group), with an aOR of 1.36 (95% CI 0.10–11.00).

**Table 5:** Outcomes of neonates born after transferring embryo with or without blastomere loss, according to multiplicity

	Singleton delivery				Multiple deliveries				All deliveries			
	Intact embryo group (*n* = 2187)	Blastomere loss group (*n* = 332)	OR (95% CI)	aOR (95% CI)^a^	Intact embryo group (*n* = 1559)	Blastomere loss group (*n* = 151)	OR (95% CI)	aOR (95% CI)^a^	Intact embryo group (*n* = 3746)	Blastomere loss group (*n* = 483)	OR (95% CI)	aOR (95% CI)^a^
	No. (%)	No. (%)			No. (%)	No. (%)			No. (%)	No. (%)		
Gestational age, Mean (SD), years	38.13 (1.76)	38.33 (1.83)			35.78 (2.04)	36.09 (2.23)			37.14 (2.22)	37.63 (2.22)		
Sex (Female)	1040 (47.6)	172 (51.8)	1.19 (0.94–1.49)	1.21 (0.95–1.55)	767 (49.2)	77 (51.0)	1.07 (0.77–1.50)	1.02 (0.72–1.44)	1807 (48.2)	249 (51.6)	1.14 (0.94–1.38)	1.13 (0.93–1.38)
Birthweight												
< 2500 g	115 (5.3)	20 (6.0)	1.16 (0.71–1.90)	1.42 (0.76–2.66)	757 (48.4)	81 (53.6)	1.23 (0.88–1.72)	1.07 (0.71–1.61)	870 (23.2)	101 (20.9)	0.89 (0.70–1.12)	0.87 (0.64–1.19)
> 4000 g	219 (10.0)	35 (10.5)	1.07 (0.73–1.56)	1.08 (0.72–1.62)	1 (0.1)	0 (0.0)	NA	NA	220 (5.9)	35 (7.3)	1.22 (0.84–1.77)	1.28 (0.86–1.92)
Birthweight for gestational age												
SGA	65 (3.0)	16 (4.8)	1.61 (0.92–2.83)	1.84 (0.99–3.37)	285 (18.3)	41 (27.2)	1.64 (1.12–2.41)	1.50 (1.00–2.25)	350 (9.3)	57 (10.6)	1.29 (0.96–1.75)	1.14 (0.83–1.56)
LGA	376 (17.2)	49 (14.8)	0.85 (0.62–1.18)	0.80 (0.57–1.13)	42 (2.7)	2 (1.3)	0.54 (0.13–2.28)	0.44 (0.10–1.90)	418 (11.2)	51 (11.8)	0.97 (0.71–1.32)	0.91 (0.66–1.26)
Apgar ≤ 7 (1 min)	30 (1.4)	7 (2.1)	1.55 (0.68–3.56)	2.52 (0.99–6.39)	43 (2.8)	5 (3.3)	1.21 (0.47–3.10)	1.80 (0.63–5.15)	73 (2.0)	12 (2.5)	1.28 (0.69–2.38)	1.39 (0.73–2.64)
Apgar ≤ 7 (5 min)	12 (0.6)	3 (0.9)	1.65 (0.46–5.88)	4.22 (0.97–18.44)	19 (1.2)	2 (1.3)	1.09 (0.25–4.73)	1.55 (0.29–8.26)	31 (0.8)	5 (1.0)	1.25 (0.48–3.24)	2.76 (0.94–8.13)
Neonatal respiratory distress												
TTN	13 (0.6)	6 (1.8)	3.07 (1.16–8.13)	6.27 (1.86–21.14)	33 (2.1)	8 (5.3)	2.60 (1.17–5.74)	5.21 (1.90–14.27)	46 (1.2)	14 (2.9)	2.38 (1.30–4.36)	5.21 (2.42–11.22)
RDS	41 (1.9)	5 (1.5)	0.82 (0.32–2.08)	1.11 (0.38–3.29)	134 (8.6)	13 (8.6)	1.04 (0.57–1.89)	0.77 (0.37–1.60)	181 (4.7)	18 (3.7)	0.80 (0.49–1.32)	0.91 (0.49–1.67)
Congenital anomaly^b^	6 (0.3)	2 (0.6)	2.20 (0.44–10.96)	1.98 (0.35–11.17)	14 (0.9)	1 (0.7)	0.73 (0.10–5.65)	0.57 (0.06–5.17)	20 (0.5)	3 (0.6)	1.16 (0.34–3.94)	1.18 (0.32–4.31)
Neonatal mortality ^c^	2 (0.1)	1 (0.3)	3.30 (0.30–36.50)	NA	6 (0.4)	0 (0.0)	NA	NA	8 (0.2)	1 (0.2)	0.97 (0.12–7.78)	1.36 (0.10–11.00)

*FET* frozen-thawed embryo transfer, *AGA* appropriate for gestational age, *SGA* small for gestational age, *LGA* large for gestational age, *TTN* transient tachypnea of the newborn, *RDS* respiratory distress syndrome, *HRT* hormone replacement therapy, *OR* odds ratio, *CI* confidence interval, *aOR* adjusted odds ratio, *NA* not accessible

^a^aOR was adjusted for gestational diabetes mellitus, hypertensive disorder, paternal smoking during pregnancy, number of previous abortion, primary infertility, preterm delivery, mode of delivery, and type of embryo cryopreservation

^b^Of the 8 singletons who had congenital anomaly, 2 in the intact embryo group had congenital heart diseases, 2 in the intact embryo group had congenital preauricular fistula, and another 2 in the intact embryo group had congenital anal atresia and hypospadias. Two singletons in the blastomere loss group had hypospadias and cheilopalatognathus. Of the 14 multiples neonates, 7 in the intact embryo group had congenital heart diseases, 2 in the intact embryo group had congenital anal atresia, 2 in the intact embryo group had multiple anomalies, the rest in the intact embryo group had spina bifida, polydactyly, and tracheobronchial malacia, respectively. One multiples neonate in the blastomere loss group had congenital heart disease

^c^Of the 3 neonatal deaths of singletons, 2 in the intact embryo group died due to respiratory distress and cerebral palsy, 1 in the blastomere loss group died due to neonatal encephalopathy. Of the 6 neonatal deaths of multiples neonates in the intact embryo group, 3 neonates died due to neonatal encephalopathy, another 3 each died due to tetralogy of Fallot, congenital anal atresia, and cerebral palsy

## Discussion

In this cohort study of 12,105 frozen cleavage-stage embryo transfer cycles, we found that embryo transfer with damaged embryos was associated with lower rates of implantation, clinical pregnancy, ongoing pregnancy, and live birth compared with intact embryo transfer. However, clinical pregnancies conceived from embryos with blastomere loss had a similar probability of progressing to live birth as those conceived from intact embryos. We also found that newborns resulting from the implantation of embryos with blastomere loss only had an increased risk of SGA in multiple pregnancies, along with an increased risk of TTN, but did not have an increased risk of other adverse neonatal outcomes compared with newborns resulting from the implantation of intact embryos.

The number of embryos transferred appears to have been influenced by the quality of the embryo when thawing, as more embryos were transferred in the blastomere loss group. However, increasing the embryo number did not fully compensate for the effect of blastomere loss. In accordance with some previous studies [17, 20, 21], blastomere loss could impair embryonic developmental potential and reduce the pregnancy rate independently of the number of embryos transferred. Our study provides more robust results with a follow-up rate of almost 98% and a large sample size. Blastomere loss is thought to be due to the blastomere lysis induced by ice crystals and exposure to the hyperosmotic environment [22]. Apart from the loss of genetic materials and disruption of cell-to-cell communication, necrotic blastomeres in the process of cryopreservation may induce a possible toxic effect on the remaining blastomeres and lead to lower viability [23]. Vitrification is commonly regarded to be superior to slow freezing in improving embryo quality and the post-warming survival rate of cleavage-stage embryos [24–26]. Consistent with other studies, we observed that vitrified embryos (92.6%; 5860/6326) were more likely to be intact than slow-frozen embryos (69.0%; 3986/5779). Since intact embryos have higher pregnancy rates and lower risks for SGA and TTN, these findings support the use of vitrification as opposed to slow-freezing methods in embryo cryopreservation.

Although individual blastomeres seem to be totipotent, an embryo with less than half of the initial number of blastomeres after cryopreservation is generally considered to be unsuitable for transfer [17]. However, the intensity of embryo damage that an embryo can survive is undefined. Accordingly, we analyzed the association between pregnancy outcome and the proportion of blastomere loss in single embryo transfer cycles (Table 3). Our data suggest that embryo competency is not adversely affected unless there is more than 25% blastomere loss. This finding is partially consistent with that of a previous study that advocated elective single embryo transfer even when an embryo had less than 25% blastomere loss [7]. Moreover, as shown in Additional file 3, the clinical pregnancy rate in the blastomere loss group gradually increased from 13.4% to 27.6% as the number of embryos transferred increased, which could partially compensate for blastomere loss. Although elective single embryo transfer has been advocated to avoid subsequent multiple pregnancy over the last few years, our findings demonstrated that the multiple pregnancy rate in the blastomere loss group was only 3.9% (75/1936) when transferring more than one embryo, which was lower than the 8.5% observed in the intact embryo group (768/9043). Our results support the policy of the single intact embryo transfer, not only to improve the pregnancy rate, but also to prevent medically induced multiple pregnancy from transferring more than one embryo with blastomere loss. Transferring more than one embryo can help to compensate for the effect of blastomere loss. However, attention must be paid to the risk of SGA and TTN in these patients.

Plausibly, blastomere loss has a negative effect on embryo development potential and decreases the implantation rate. Some important processes, including embryonic genome activation, genomic imprint maintenance, and methylation reprogramming of non-imprinted genes, occur in the preimplantation stage [27, 28]. The reduction in viable embryonic material and disruption of cell-to-cell communication might play a role [27]. However, our study demonstrates that blastomere loss does not result in an increased miscarriage rate among clinical pregnancies. This finding is consistent with the results of a previous retrospective study reporting that there is no relationship between embryo quality and the abortion rate [29].

Our study addresses the long-term safety of blastomere loss in terms of neonatal outcome, a topic which has not been previously addressed. Several outcome measures, including birth weight for gestational age, RDS, Apgar score, congenital anomalies, and neonatal mortality, were similar between both groups. The increased rate of cesarean section in the blastomere loss group may partially explain the association between blastomere loss and TTN. Our observation of similar frequencies of adverse outcomes among blastomere loss and intact embryo neonates validates the value and safety of transferring an embryo with blastomere loss.

PGD or preimplantation genetic screening are typically performed with a highly invasive biopsy procedure that involves aspirating one or two blastomeres from day 3 post-fertilization embryos, at the 6–8 cell stage. A previous study showed that the biopsy did not cause intra-uterine growth restriction or low birth weight compared with spontaneous pregnancy [30]. The present results (Table 3) reveal that embryos may not be vulnerable when blastomere loss is less than 25%, as the clinical pregnancy rate and live birth rate are not affected, thus further strengthening the safety of blastomere biopsy. However, whether spontaneous blastomere loss is comparable to cell loss by PGD in terms of its effect on embryo development remains uncertain.

In the present study, we observed a trend toward an association between blastomere loss and SGA in multiples. A similar trend was observed in singletons. A possible explanation for the increased risk of SGA may that blastomere loss may result in impaired placental implantation or function. However, a limitation of the study is that placental weight was not documented. Major congenital anomalies and neonatal mortality were not found to be associated with blastomere loss, which suggests the relative safety of embryos with blastomere loss. This risk raises some concerns that need to be addressed in future studies of embryo transfer with larger sample sizes and that take into consideration placental characteristics.

This is the first multicenter cohort with a large sample size to quantify the adverse outcomes of neonates born from embryos with blastomere loss compared with those born from intact embryos. The findings of the present study provide valuable information to obstetricians and pediatricians managing pregnancies and newborns derived from embryos with blastomere loss. As a multicenter cohort design, selection bias was minimized. The large sample size helped to achieve sufficient power to assess the clinical efficiency of embryo transfer with damaged embryos, as well as the risk of adverse neonatal outcomes.

However, the study still has some limitations. Due to the low incidence of some outcomes including congenital anomalies and neonatal mortality, these variables have wide CIs. Furthermore, we only followed up the major birth defects for 1 month after delivery and recorded the malformations in general appearance, the cardiovascular system, or the central nervous system, which could be detected by ultrasonography. Some cases of occult congenital heart disease, as well as undetected cases with chromosomal disorders, molecular diseases, and metabolic diseases that were not diagnosed within the first month of life, may have existed. Loss to follow-up was attributed to changes in domicile and was an unlikely source of bias. We did not collect information on the long-term follow-up of growth and development.

## Conclusions

In summary, the transfer of embryos with blastomere loss induced by embryo cryopreservation is associated with reduced rates of conception but is not associated with an increased risk of preterm birth, perinatal mortality, or congenital anomalies. However, pregnancies conceived from embryos with blastomere loss may be at increased risk of SGA. Evidence for this effect is strongest among women with multiple births. Long-term follow-up studies are required to assess the possible effects on child growth and development.

## Additional files

Additional file 1: Pregnancy outcomes following the transfer of two or three embryos. (DOCX 137 kb)

Additional file 2: Pregnancy outcomes analysis stratified according to the type of cryopreservation. (DOCX 137 kb)

Additional file 3: Comparison of association between clinical pregnancy rate and number of transferred embryo of each group. (DOCX 223 kb)

Additional file 4: Comparison of association between live birth rate and number of transferred embryo of each group. (DOCX 258 kb)

## Abbreviations

aOR: adjusted odds ratio
CI: confidential interval
FET: frozen-thawed embryo transfer
IVF: in vitro fertilization
OR: odds ratio
PGD: preimplantation genetic diagnosis
RDS: respiratory distress syndrome
SGA: small for gestational age
TTN: transient tachypnea of the newborn
