# Supplementary material for: Outcomes of neonates born following transfers of frozen-thawed cleavage-stage embryos with blastomere loss: a prospective, multicenter, cohort study
Source: BMC Med. 2018 Jun 19;16:96. doi: 10.1186/s12916-018-1077-8 (PMC6006714; doi:10.1186/s12916-018-1077-8)
Supplement: Supplementary file 1 — Pregnancy outcomes following the transfer of two or three embryos. (DOCX 137 kb) [file 12916_2018_1077_MOESM1_ESM.docx]

| **Additional file 1: Pregnancy outcomes following the transfer of two or three embryos** | | | | | | | |  |
| --- | --- | --- | --- | --- | --- | --- | --- | --- |
|  | **Transfer of two embryos** | | |  | **Transfer of three embryos** | | | |
|  | **Intact embryo (N=6679)** | **Blastomere Loss (N=1015)** | ***p* value** |  | **Intact embryo (N=2364)** | **Blastomere Loss (N=924)** | ***p* value** | |
| **Total No. of embryos transferred** | 13358 | 2030 |  |  | 7092 | 2772 |  | |
| **Embryo Implantation rate (n, %)** ^a^ | 3108 (23.3) | 244 (12.0) | <0.001 |  | 1347 (19.0) | 344 (12.4) | <0.001 | |
| **Chemical pregnancies (n, %)** ^b^ | 2923 (43.8) | 234 (23.1) | <0.001 |  | 1042 (44.1) | 288 (31.2) | <0.001 | |
| **Clinical pregnancies (n, %)** ^c^ | 2496 (37.4) | 201 (19.8) | <0.001 |  | 969 (41.0) | 255 (27.6) | <0.001 | |
| **Ongoing pregnancies (n, %)** ^d^ | 2163 (32.4) | 164 (16.2) | <0.001 |  | 825 (34.9) | 221 (23.9) | <0.001 | |
| **Live birth (n, % per embryo transfer cycle)** ^e^ | 2043 (30.6) | 157 (15.5) | <0.001 |  | 805 (34.1) | 215 (23.3) | <0.001 | |
| **Live birth (n, % per clinical pregnancy)** ^f^ | 2043 (81.9) | 157 (78.1) | 0.188 |  | 805 (83.1) | 215 (84.3) | 0.637 | |
| Singleton (% per live birth) | 1547 (75.7) | 138 (87.9) | 0.001 |  | 533 (66.2) | 160 (74.4) | 0.066 | |
| Twins (% per live birth) | 495 (24.2) | 19 (12.1) |  |  | 264 (32.8) | 54 (25.1) |  |  |
| Triplets (% per live birth) | 1 (0.1) | 0 (0.0) |  |  | 8 (1.0) | 1 (0.5) |  |  |
| **Ectopic pregnancies (n, %)** | 79 (1.2) | 7 (0.7) | 0.164 |  | 18 (0.8) | 12 (1.3) | 0.145 | |
| **Early miscarriages (n, % per clinical pregnancy)** | 333 (13.3) | 37 (18.4) | 0.057 |  | 143 (14.8) | 34 (13.3) | 0.565 | |
| **Stillbirth (n, % per clinical pregnancy)** | 5 (0.2) | 0 (0.0) | 1.000 |  | 8 (0.8) | 2 (0.8) | 1.000 | |
| **Pregnancy termination due to fetal anomaly (n, % per clinical pregnancy)** ^g^ | 9 (0.4) | 1 (0.5) | 0.540 |  | 1 (0.1) | 1 (0.4) | 0.373 | |
| ^a^ The implantation was defined as an observation of gestational sacs by ultrasound. The implantation rate was defined as the number of gestational sac divided by the number of embryo transferred. | | | | | | | |  |
| ^b^ Chemical pregnancy was defined as an elevated serum β-hCG level of more than 10 mIU/ml. Chemical pregnancy rate was defined as the number of chemical pregnancy divided by the number of embryo transfer cycle for each group. | | | | | | | |  |
| ^c^ Clinical pregnancy was defined as a pregnancy documented by ultrasound at 6-8 gestational weeks that showed a gestational sac inside the uterus. Clinical pregnancy rate was defined as the number of clinical pregnancy divided by the number of embryo transfer cycle for each group. | | | | | | | |  |
| ^d^ Ongoing pregnancy was defined as a pregnancy documented by ultrasound at 12 gestational weeks that showed the presence of fetal heartbeat. Ongoing pregnancy rate was defined as the number of ongoing pregnancy divided by the number of embryo transfer cycle for each group. | | | | | | | |  |
| ^e^ Live birth was defined as the delivery of one or more infants with any signs of life after 28 gestational weeks. Live birth rate (% per embryo transfer cycle) was defined as the number of live birth divided by the number of embryo transfer cycle for each group. | | | | | | | |  |
| ^f^ Live birth rate (% per clinical pregnancy) was defined as the number of live birth divided by the number of clinical pregnancy for each group. | | | | | | | |  |
| ^g^ Of the 9 patients who terminated the pregnancy in intact embryo group of two embryos transfer, 3 were diagnosed as chromosome anomalies, 2 were never system development disorder, 2 were multiple malformation, the rest were umbilical hernia, and bilateral renal agenesis respectively. One in intact embryo group of three embryo transfer terminated pregnancy due to congenital heart disease. Both 2 patients in blastomere loss group terminated pregnancy due to the limb deformity. | | | | | | | |  |
