# Supplementary material for: Outcomes of neonates born following transfers of frozen-thawed cleavage-stage embryos with blastomere loss: a prospective, multicenter, cohort study
Source: BMC Med. 2018 Jun 19;16:96. doi: 10.1186/s12916-018-1077-8 (PMC6006714; doi:10.1186/s12916-018-1077-8)

**Additional file 3: Comparison of association between clinical pregnancy rate and number of transferred embryo of each group**


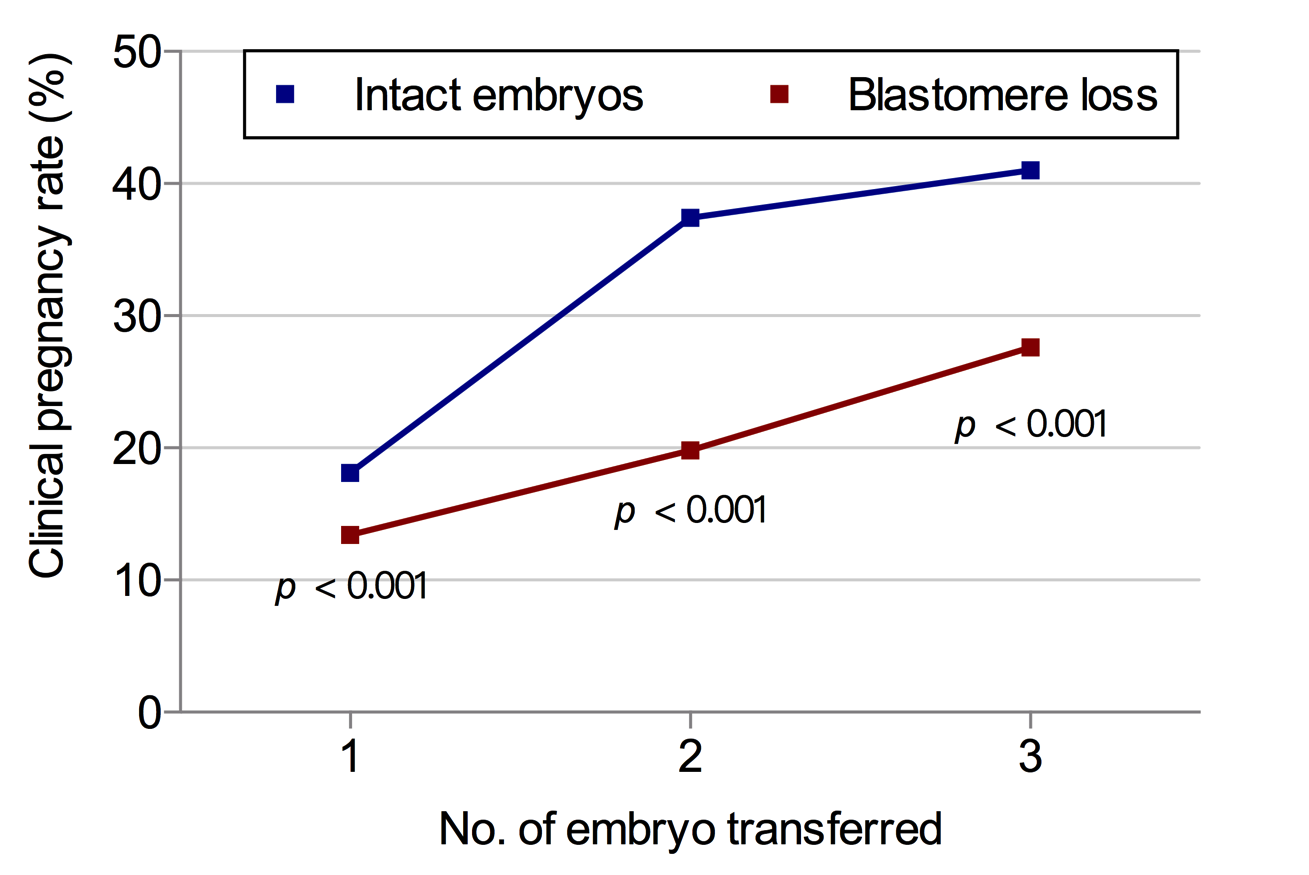

Supplement: Supplementary file 3 — Comparison of association between clinical pregnancy rate and number of transferred embryo of each group. (DOCX 223 kb) [file 12916_2018_1077_MOESM3_ESM.docx]
