# Supplementary material for: Outcomes of neonates born following transfers of frozen-thawed cleavage-stage embryos with blastomere loss: a prospective, multicenter, cohort study
Source: BMC Med. 2018 Jun 19;16:96. doi: 10.1186/s12916-018-1077-8 (PMC6006714; doi:10.1186/s12916-018-1077-8)
Supplement: Supplementary file 4 — Comparison of association between live birth rate and number of transferred embryo of each group. (DOCX 258 kb) [file 12916_2018_1077_MOESM4_ESM.docx]

**Additional file 4: Comparison of association between live birth rate and number of transferred embryo of each group**


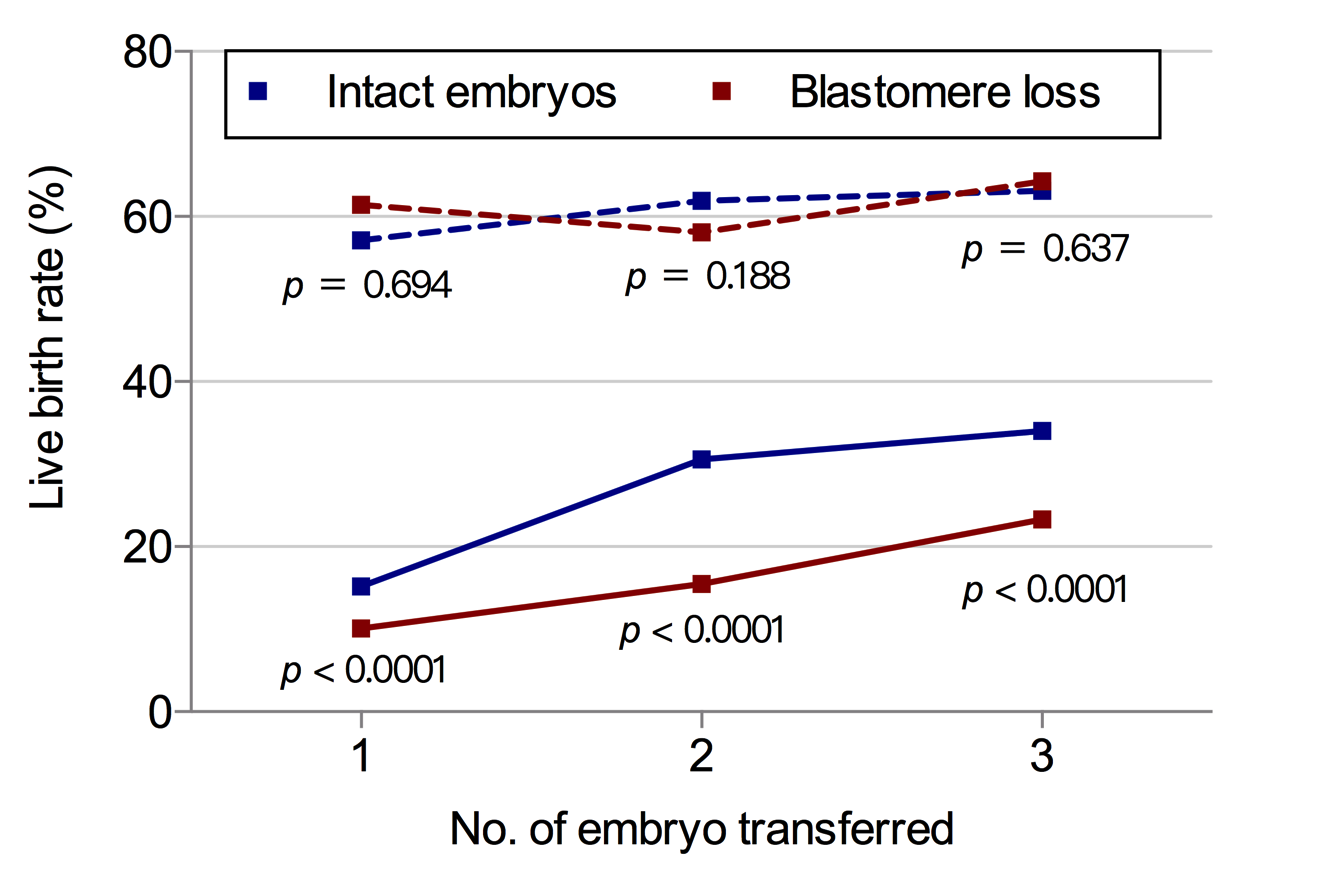


Figure legend

Dotted line, live birth rate per clinical pregnancy; solid line, live birth rate per embryo transfer cycle
